# An observational study: The utility of perfusion index as a discharge criterion for pain assessment in the postanesthesia care unit

**DOI:** 10.1371/journal.pone.0197630

**Published:** 2018-05-16

**Authors:** Chun-Lin Chu, Yi-You Huang, Ying-Hou Chen, Ling-Ping Lai, Huei-Ming Yeh

**Affiliations:** 1 Institute of Biomedical Engineering National Taiwan University, Taipei, Taiwan; 2 Department of Anesthesiology, National Taiwan University Hospital Yun-Lin Branch, Yun-Lin, Taiwan; 3 Department of Cardiology, National Taiwan University Hospital, Taipei, Taiwan; 4 Department of Anesthesiology, National Taiwan University Hospital, Taipei, Taiwan; National Taiwan University, School of Dentistry, TAIWAN

## Abstract

Acute post-operative pain can remain untreated if patients cannot express themselves. The perfusion index (PI) may decrease when pain activates sympathetic tone and may increase after analgesics are administered. We evaluated if the perfusion index is a feasible indicator for objectively assessing pain relief in the postanesthesia care unit (PACU) and calculated the changes in PI measurements at the time of discharge from the PACU relative to baseline PI measurements to examine if the PI is a useful criterion for discharging patients from the postanesthesia care unit. This retrospective observational study enrolled female patients who were admitted for gynecological or general surgery. The patients received general anesthesia and were admitted to the postanesthesia care unit. The PI, visual analogue scale (VAS) score, heart rate, and blood pressure were recorded before and after administration of intravenous morphine. Changes in these parameters before and after analgesics were administered and the difference of these parameters between age and BMI subgroups were compared. The correlation between the PI and VAS score, ΔPI and ΔVAS, and %ΔPI and %ΔVAS were also evaluated. The percentage change in ΔPI (P9-T0/T0) of the patients at the time of discharge from the postanesthesia care unit relative to baseline PI measurements was calculated. Eighty patients were enrolled, and there were 123 instances during which analgesia was required. Heart rate, PI, and VAS score were significantly different before and after analgesics were administered (p < 0.0001). The difference of parameters between age and BMI subgroups were not significant. The correlation between the PI and VAS score, ΔPI and ΔVAS, and the percentage change in ΔPI and ΔVAS showed weak correlations in age, BMI subgroups, and all measurements. The baseline PI and the PI when arriving at and when being discharged from the postanesthesia care unit were significantly different (p < 0.01). The mean percentage change in Δ PI at the time of discharge from the PACU was 66.2%, and the 99% confidence interval was 12.2%~120.3%. The perfusion index was increased, and the VAS score was decreased significantly after analgesics were administered, but the correlation was weak in each subgroup. The VAS score is a subjective and psychometric parameter. The PI increased when partial pain relief was achieved after morphine was administered but did not reflect pain intensity or changes in the VAS score regardless of age or BMI. A percentage change in ΔPI at the time of discharge from the PACU relative to baseline PI measurements of greater than 12% can be used as a supplemental objective discharge criterion for pain assessment in the postanesthesia care unit.

## Introduction

Pain is a subjective sensation that can negatively impact psychological and physiological wellbeing. It can stimulate the sympathetic nerve system and release stress hormones leading to increased oxygen consumption and even resulting in myocardial ischemia in serious cases [1]. Reducing pain during surgery and preventing and managing pain post-operatively is of crucial importance in the perioperative period [2], and patients care greatly about this issue.

Caregivers prescribe analgesics based on the extent of surgery and their experiences in the postanesthesia care unit (PACU). They also use the visual analogue scale (VAS) score or numeric rating scale (NRS) to subjectively estimate pain severity according to patients’ facial expressions and self-reported pain scores. Then, they select the appropriate analgesic and evaluate the response to treatment. However, there are still cases of pain that remain unresolved or undertreated because the patients were unable to express themselves adequately, such as in patients with mental retardation or dementia. Several studies regarding objective pain assessment tools, such as the surgical stress index during anesthesia [3] and the analgesic nociception index in the postanesthesia care unit [4], to supplement subjective feedback have been reported.

The perfusion index (PI), which is the ratio between the variable pulsatile (DC) and nonpulsatile (AC) signals, is an indirect and noninvasive measurement of peripheral perfusion [5]. It is calculated by means of pulse oximetry by expressing the pulsatile signal (during arterial flow) as a percentage of the nonpulsatile signal (AC/DC X 100), both of which are derived from the amount of infrared (940 nm) light absorbed. The PI may decrease due to increased vasomotor tone and the contraction of peripheral blood vessels when the sympathetic nervous system is activated by pain [6]. The PI may also increase when pain is relieved by the use of adequate analgesics [7]. In this study, we correlated the perfusion indices and visual analogue scale scores of patients to test if the perfusion index is a useful marker for objectively assessing pain relief in the PACU. We also compared the change in PI (ΔPI) at the time of discharge from the postanesthesia care unit with baseline PI measurements to evaluate if the PI is a useful criterion for discharging patients from the postanesthesia care unit.

## Materials and methods

### Ethics declaration

The protocol used in this study was approved by the Institutional Ethics Review Board of National Taiwan University Hospital (Registry Number: 201604074RINB), and informed consent was waived based on its retrospective design. This study was carried out according to the International Conference on Harmonisation (ICH)/WHO Good Clinical Practice (GCP) guidelines and conformed to the principles outlined in the Declaration of Helsinki.

#### Study design and subjects

This was an observational, retrospective, and single-center study. We reviewed medical records and enrolled female patients aged from 20 to 80 years old with an ASA class of I~III who were scheduled for gynecologic or general surgery and were admitted to the postanesthesia unit at National Taiwan University Hospital between November 2015 and May 2016. The exclusion criteria included patients who had unstable vital signs, those who were admitted as emergency cases, those who were intubated, those who were medicated with sedative or vasoactive agents, those who had been diagnosed with peripheral occlusive artery disease, and those who were admitted to the intensive care unit. The patients were monitored with automated noninvasive blood pressure on one arm, a 3-lead electrocardiogram, and a Masimo Radical 7 pulse oximeter probe (Masimo Crop, Irvine, California) on the contralateral index finger for continuous monitoring until discharge from the postanesthesia care unit. The room temperature was maintained at 22°C, and general anesthesia was induced with fentanyl at 1~2 μg/kg, propofol at 1.5~2 mg/kg, and cisatracurium at 0.2 mg/kg. After tracheal intubation, inhalational sevoflurane or desflurane was maintained at a concentration of 1 to 1.3 MAC and adjusted according to the patient’s vital signs. After surgery was finished, patients were administered reversal agents (2.5 mg of neostigmine and 0.4 mg of glycopyrrolate 0.4 mg) when spontaneously breathing was regained, and then the patients were sent to the postanesthesia care unit for observation. According to chart record, baseline data (T0) including perfusion index, temperature, heart rate, mean blood pressure, and SpO2 were recorded 5 minutes after entering the operating room. Postanesthetic data (P0) were recorded when the patients were admitted to the PACU after surgery. The perfusion index and visual analogue scale (VAS) score were recorded when the patients regained consciousness and asked for analgesics for the first time (P1), the second time (P2), and the third time (P3). The PI and VAS were recorded as P10, P20, and P30, 5 minutes after 3 mg of morphine was administered intravenously. Before discharge from the postanesthesia care unit, the PI and VAS were recorded at time point P9. The primary hypothesis of our study was that perfusion index is correlated with VAS, and the secondary hypothesis was that the percentage change of the perfusion index would be useful as a discharge criterion for assessing the wellbeing of the patients.

### Statistical analysis

We used GraphPad Prism 6 to perform the statistical analysis. Continuous data are shown as the mean ± SD, and categorical data are shown as percentages. The Shapiro-Wilk test was used to test the normality of the distribution. We used the Wilcoxon signed-rank test or the paired *t*-test to separately compare the differences between repeated measurements of non-parametric and normally distributed data. The Friedman test was also used to compare the PI measured at different time points. Patients were divided into age subgroups and BMI subgroups and the difference of parameters before and after analgesic administration between these subgroups were compared by Kruskal–Wallis test. Correlation between the PI and the VAS was tested by Persons’ correlation coefficient in age, BMI subgroups, and all measurements. To reduce the bias of individual variation in perfusion index, we also evaluate the correlation between the ΔPI (PI after analgesic administration—PI before analgesic administration) and ΔVAS (VAS after analgesic administration—VAS before analgesic administration), and the percentage change in ΔPI (ΔPI/PI before analgesic administration) and the percentage change in ΔVAS (ΔVAS/VAS before analgesic administration). The PI measurements at the time when the patients met the discharge criteria were compared with the baseline PI measurements. The percentage change in Δ PI (P9-P0/P0) at the time of discharge from the postanesthesia care unit was calculated, and 99% confidence intervals were constructed to evaluate the use of this measure as a discharge criterion, along with VAS scores < 3. All the tests were 2-tailed, and p < 0.01 was considered statistically significant.

## Results

**Fig 1:**
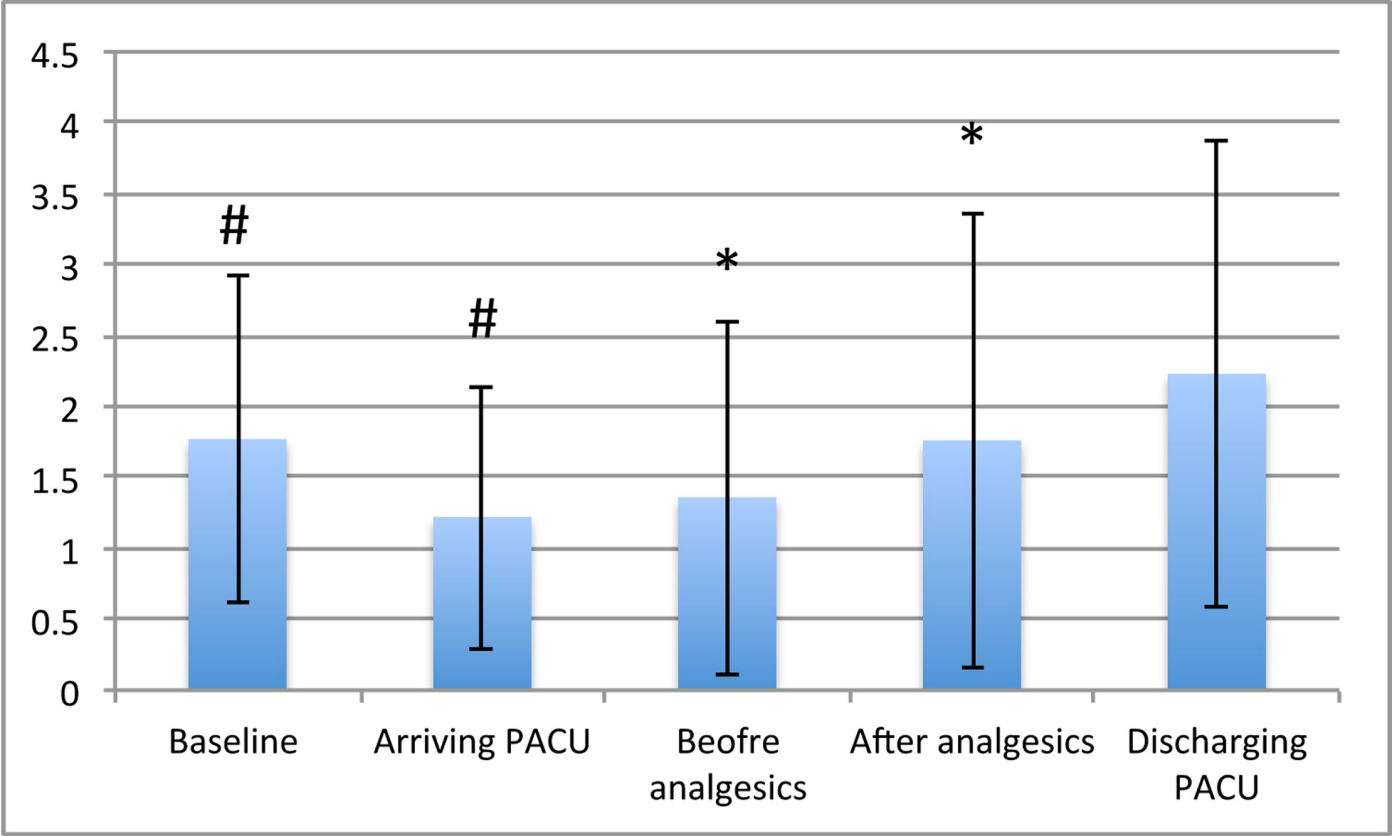
Perfusion index changes at different time points. The perfusion index at the time of arrival to the postanesthesia care unit (PACU) was significantly different from the baseline PI. Differences in the PI between before and after the administration of analgesics also existed. There was no difference between the baseline PI and the PI at the time of discharge from the PACU.

One hundred three patients were screened, and 80 patients met the inclusion criteria. The demographic data are shown in Table 1. There were 64 and 16 female patients receiving gynecologic and general surgery, respectively. The average morphine consumption in the postanesthesia care unit was 4.5 mg, with an average number of analgesic requests of 1.5. The perfusion index was measured at different time point and is expressed as the mean ± standard deviation in Fig 1. There was significant difference between the baseline PI (T0) and the PI at the time of arrival to postanesthesia care unit (P0) (p<0.001). The PI at the time of discharge from the postanesthesia care unit (P9) was not significantly different from the baseline PI (T0) (p = 0.1362).

**Table 1:** Demographic data.

	N = 80
**Age (years)**	45.7 ± 13.2
**20~40**	28
**40~60**	39
**above 60**	13
**Female Gender**	80 (100%)
**Body Mass Index (kg/m**^**2**^**)**	25.2 ± 2.8
**Normal Weight (18.5~24.9)**	33
**Over Weight (25~29.9)**	42
**Obesity (>30)**	5
**ASA classification (I/II/III)**	7/55/18
**Intraoperative intravenous fluid administration**	892 ± 225
**Post-operative morphine usage**	4.5 ± 1.7
**Gynecological surgery vs. General surgery**	64 /16

In total, there were 123 requests for analgesic medication. The PI before analgesic administration (P1, P2, and P3) and the PI after analgesic administration (P10, P20, and P30) were significantly different (p < 0.0001). The VAS score and heart rate before and after analgesic administration also significantly changed (p < 0.0001) (Table 2). The PI and the VAS score before and after analgesic administration, the ΔPI (PI after analgesic administration—PI before analgesic administration) and the ΔVAS (VAS after analgesic administration—VAS before analgesic administration), and the percentage change in ΔPI (ΔPI/PI before analgesic administration) and the percentage change in ΔVAS (ΔVAS/VAS before analgesic administration) were no significant difference between age and BMI subgroups as showed in S1 Table and were all weakly correlated (Table 3). There was significant correlation between PI and VAS before and after analgesic administration (r = 0.742, 0.778 separately) in obesity group (BMI > 30 kg/m^2^). All the patients had a VAS score < 3 with regard to their wellbeing when they were discharged from the postanesthesia care unit. The mean percentage change in ΔPI (P9-P0/P0) at the time of discharge from the postanesthesia care unit was 66.2%, and the 99% confidence interval was 12.2% ~ 120.3%.

**Table 2:** Changes in parameters before and after the administration of analgesics.

n = 123	Before analgesics	After analgesics	p value
**Mean arterial pressure**	87.7 ± 12.8	85.2 ± 12.4	0.0003
**Heart Rate**	72.4 ± 12.3	70.0 ± 11.6	<0.0001
**Perfusion index**	1.3 ± 1.2	1.7 ± 1.6	<0.0001
**Visual Analogue Scale Score**	6.6 ± 2.0	4.7 ± 2.3	<0.0001

**Table 3:** Correlation between PI and VAS, ΔPI and ΔVAS, and percentage changes in ΔPI and ΔVAS in age or BMI subgroups and all measurement.

Pearson’s coefficient	PI vs. VAS (pre-)	PI vs. VAS (post-)	ΔPI vs. ΔVAS	% ΔPI vs. % ΔVAS
**Age (y/o)**				
**20–40 (46)**	0.186	-0.087	0.210	0.185
**40–60 (53)**	-0.312	-0.346	0.235	0.181
**> 60 (24)**	-0.356	-0.362	0.113	0.039
**BMI (kg/m**^**2**^**)**				
**18.5~24.9 (41)**	-0.425	-0.428	0.253	0.154
**25~29.9 (74)**	0.030	-0.186	0.224	0.195
**>30 (8)**	0.742*	0.778*	-0.506	-0.586
**All measurement**				
**Total (123)**	-0.0624	-0.1986	0.0845	0.1153

ΔPI: PI before analgesic administration–PI after analgesic administration; % ΔPI: (PI before analgesic administration–PI after analgesic administration)/PI before analgesic

* p value < 0.05

## Discussion

The perfusion index (PI) is derived from plethysmography and is a non-invasive and convenient tool for evaluating peripheral perfusion. It can be used in routine anesthetic practice and can help anesthetists to make decisions based on its characteristics. Several studies were carried out in recent years because the perfusion index was thought to have become more stable and reliable [8]. It has been used to predict hypotension after spinal anesthesia during caesarean delivery [9, 10] and as an early indicator of successful nerve block or sympathectomy [11, 12]. It is also correlated with anesthetic depth [13] and can detect stress responses during anesthesia [7]. A decreased PI was associated with changes in position in critically ill non-intubated patients, and the correlation between changes in the PI and changes in BPS-NI values was positive [6]. Perfusion index as a tool for monitoring acute post-operative pain has been surveyed, but its correlation with visual analogue scale scores was not statistically significant [14].

In our study, the PI at the time of arrival to the postanesthesia care unit was lower than the baseline PI as a result of acute post-operative pain. There was a significant change in heart rate, PI, and VAS before and after the administration of intravenous morphine. Pain can stimulate a sympathetic reaction that increases heart rate and results in peripheral vasoconstriction, leading to a decreased PI. Morphine is an effective and safe analgesic medication for managing acute post-operative pain that can be given incrementally [15]. When acute post-operative pain was well managed, the PI increased, and the VAS score decreased significantly. When patients requested more analgesia and reported a VAS score of more than 5, the PI increased further after the second administration of intravenous morphine.

Like in a previous study, the correlation between the perfusion index and the VAS score was not established in our study. The ΔPI and ΔVAS were not significantly correlated, and the percentage changes in ΔPI and ΔVAS were also not significantly different. There was still no significant correlation in age and BMI subgroups with exception of PI and VAS before and after analgesic administration in obesity group (BMI > 30 kg/m^2^) This can be explained because the VAS score is subjective and is based on psychometric properties. Patients may still report higher VAS scores when they suffer from poor emotional and psychological wellbeing even if analgesics are administered. The PI increased due to partial pain relief when analgesics were administered but did not reflect the VAS scores that the patients subjectively reported. This irrelevant phenomenon existed regardless of age and BMI. The only significant correlation between PI and VAS before and after analgesic was due to rather few measurements in morbid obesity group.

The criteria for discharge from the postanesthesia care unit included full recovery of consciousness, adequate respiration, and stable blood pressure and heart rate. Adequate pain control with a VAS score < 3 was also one of the criteria. All the patients who were discharged from the postanesthesia care unit were treated and had VAS scores < 3 in this study. We compared the patients’ baseline PI measurements with the PI at the time of discharge from the PACU and found that there was no significant difference. The mean of the percentage change in ΔPI was 66.2% with a 99% confidence interval of 12.2% ~ 120.3%. Individual baseline variation in PI measurements has been lessened by using this parameter, but there is still wide range of data distribution. Most patients discharged from the PACU were in good condition and reported VAS scores < 3 with the percentage change in ΔPI at the time of discharge from the postanesthesia care unit greater than 12%. As the above result shows, the percentage change in ΔPI can be used as a supplemental objective pain evaluation tool in the postanesthesia care unit if the patient is unconscious and cannot properly report a VAS score.

The major limitation of our study is that we included only female patients who underwent gynecological or general surgery in order to achieve homogeneity. However, older women showed less significant changes in PI as measured by electrical stimulation [16]. This may have lessened the significance of the changes in perfusion index and VAS and have led to negative results. Patients who were using patient-controlled analgesia were not included in our study and who failed to meet PACU discharge criterion were not included as contrast group. A large-scale study including a larger range of surgeries and patient groups is needed to explore the utility of perfusion index measurements in anesthetic management.

We used perfusion index as a supplemental tool for pain assessment in the postanesthesia care unit. PI values increased when intravenous analgesics were administered, but the correlation of the PI with VAS was poor due to the subjectivity of VAS. We also used the percentage change in ΔPI at the time of discharge from the PACU as a discharge criterion to lessen inter-individual variation. We came to the conclusion that a percentage change in the perfusion index at the time of discharge from the postanesthesia care unit relative to baseline PI measurements of more than 12% can be used as a supplemental objective discharge criterion for pain assessment in the postanesthesia care unit.

## Supporting information

S1 Table Difference of parameters between age and BMI groups. (DOCX)

S2 Table STROBE statement checklist. (DOCX)

S3 Table Protocol of study. (DOCX)
