# Supplementary material for: An observational study: The utility of perfusion index as a discharge criterion for pain assessment in the postanesthesia care unit
Source: PLoS One. 2018 May 16;13(5):e0197630. doi: 10.1371/journal.pone.0197630 (PMC5955537; doi:10.1371/journal.pone.0197630)
Supplement: S1 Table — (DOCX) [file pone.0197630.s001.docx]

**S1 Table. Difference of parameters between age and BMI subgroups**

| n=123 | **PI (pre-)** | **VAS(pre-)** | **PI(post-)** | **VAS(post-)** | **ΔPI** | **ΔVAS** | **%ΔPI** | **%ΔVAS** |
| --- | --- | --- | --- | --- | --- | --- | --- | --- |
| **Age ( y/o)** |  |  |  |  |  |  |  |  |
| 20-40 (46) | 1.58 | 7.1 | 1.92 | 5.2 | 0.34 | 1.89 | 27.5 | 26.9 |
| 40-60 (53) | 1.24 | 6.4 | 1.59 | 4.7 | 0.35 | 1.71 | 42.0 | 27.2 |
| Above (24) | 2.19 | 6.4 | 1.99 | 4.0 | 0.59 | 2 | 53.3 | 30.8 |
| p value | 0.234 | 0.178 | 0.54 | 0.12 | 0.45 | 0.78 | 0.3 | 0.8 |
| **BMI (kg/m^2^)** |  |  |  |  |  |  |  |  |
| 18.5~24.9 (41) | 1.35 | 6.3 | 1.89 | 4.5 | 0.53 | 1.79 | 56.5 | 29.5 |
| 25~29.9 (74) | 1.27 | 6.8 | 1.64 | 4.9 | 0.36 | 1.86 | 37.5 | 26.9 |
| >30 (8) | 1.57 | 6.7 | 1.85 | 5.2 | 0.27 | 1.5 | 12.3 | 22.8 |
| p value | 0.756 | 0.36 | 0.684 | 0.523 | 0.524 | 0.861 | 0.258 | 0.75 |
