# Supplementary material for: An observational study: The utility of perfusion index as a discharge criterion for pain assessment in the postanesthesia care unit
Source: PLoS One. 2018 May 16;13(5):e0197630. doi: 10.1371/journal.pone.0197630 (PMC5955537; doi:10.1371/journal.pone.0197630)
Supplement: S3 Table — (DOCX) [file pone.0197630.s003.docx]

Study design

This is a retrospective, observational study to review medical record perioperatively

Objective

Female patients aged between 20-80 y/o with ASA classification I~III came to receive scheduled gynecologic or general surgery during November 2015 and May 2016

Inclusion criteria

1.Female patients

2. Scheduled gynecologic or general surgery

3. age between 20-80

4. ASA classification I~II

Exclusion criteria

1. Vital sign unstable
2. Emergency surgery
3. Patient who was intubated
4. Patients under inotropic and sedative medication
5. Patients with diagnosis of peripheral arterial occlusion disease
6. Patients who was under oxygen therapy
7. Patients who was admitted to intensive care unit
8. Use vasoactive or vasodilator periopertively

Method

We reviewed anesthetic record and post-anesthetic room record to enroll patients. We record patients’ demographic data and anesthetic method with de-linkage policy and record parameters at different time points as following :

T0 Baseline when entering operation room

P0 When arrival at PACU

P1 Patients ask for analgesia for first time

P10 5 minutes after giving analgesia

P2 Patients ask for analgesia for second time

P20 5 minus after giving analgesia

P3 Patients ask for analgesia for third time

P30 5 minus after giving analgesia

P9 When patient check out PACU

103 patients were screened according to inclusion criteria. 13 patients who met exclusion criteria and 10 patients with incomplete record were excluded. There were 80 patients enrolled in this study.
